# Optimal Methods of RTK-GPS/Accelerometer Integration to Monitor the Displacement of Structures

**DOI:** 10.3390/s120101014

**Published:** 2012-01-17

**Authors:** Jinsang Hwang, Hongsik Yun, Sun-Kyu Park, Dongha Lee, Sungnam Hong

**Affiliations:** Department of Civil, Architectural & Environmental Engineering, Sungkyunkwan University, Suwon 440-746, Korea; E-Mails: gpsboy@skku.edu (J.H.); yoonhs@skku.edu (H.Y.); skpark@skku.edu (S.-K.P.)

**Keywords:** RTK-GPS, accelerometer, integration, displacement monitoring

## Abstract

The accurate measurement of diverse displacements of structures is an important index for the evaluation of a structure’s safety. In this study, a comparative analysis was conducted to determine the integrated RTK-GPS/accelerometer method that can provide the most precise structure displacement measurements. For this purpose, three methods of calculating the dynamic displacements from the acceleration data were comparatively analyzed. In addition, two methods of determining dynamic, static, and quasi-static displacements by integrating the displacements measured from the RTK-GPS system and the accelerometer were also comparatively analyzed. To ensure precise comparison results, a cantilever beam was manufactured onto which diverse types of displacements were generated to evaluate the measurement accuracy by method. Linear variable differential transformer (LVDT) measurements were used as references for the evaluation to ensure accuracy. The study results showed that the most suitable method of measuring the dynamic displacement with the accelerometer was to calculate the displacement by filtering and double-integrating the acceleration data using the FIR band-pass filter. The integration method that uses frequency-based displacement extraction was most appropriate for the integrated RTK-GPS/accelerometer method of comprehensively measuring the dynamic, static, and quasi-static displacements.

## 1. Introduction

With the development of design technology, civil structures are increasingly being designed with thinner, lighter, and more flexible designs. The volume of construction materials is also decreasing, and structures are gaining economic and aesthetic advantages. Most civil structures, however, are constantly exposed to diverse natural and environmental conditions, including strong winds, earthquakes, and tsunamis, and therefore, to abnormal ultimate loads. Abnormal ultimate loading may even cause structures to collapse. An example is the Tacoma Narrows Bridge, which was opened in 1940. This bridge was the third longest suspension bridge in the US, and it collapsed within months of opening due to unexpected strong winds, which were not considered in the design stage. Therefore, it is important to monitor and evaluate the structural integrity of the main infrastructure. The structural integrity of many items can be evaluated, but displacement is generally used. This is because short-term and long-term displacements are indicators of structural behavior, and structural integrity can be evaluated in diverse ways using these displacement values. Accordingly, precise displacement measurement is required to ensure the reliable evaluation of a structure’s condition [1–3].

The accelerometer is a widely used instrument for measuring structural displacements [4,5]. When the displacement is acquired by processing the acceleration data from the accelerometer, the dynamic displacement, among the displacements in the structure, is very precise. As low-frequency signals are removed, however, and when the noise in the measured acceleration data is removed, it is difficult to measure the precise static and quasi-static displacements.

The real-time kinematic global positioning system (RTK-GPS) is recognized as efficient for measuring static and quasi-static displacements, due to its high accuracy (about 1 cm error for temporary measurement and about 1 mm error for long-term measurement) and its ability to measure the 3D absolute coordinates based on the terrestrial reference frame [6–8]. RTK-GPS has limited use, however, due to its inadequacy in measuring frequency, the dependence of its accuracy on the GPS satellite signal reception environment, and its poor properties with respect to the error sources, including the multipath errors. Tamura *et al.* [9] reported that RTK-GPS can only measure the displacements of structures with a natural frequency of 2 Hz or less and a displacement of 2 cm or more. This means that RTK-GPS cannot measure the high-frequency dynamic displacement of structures. Many studies have been conducted to ensure precise displacement measurement using the advantages of both the accelerometer and RTK-GPS [1,10–12]. Although there are diverse methods of processing the acceleration data and of acceleration/RTK-GPS data integration, the accuracy of these methods has not yet been evaluated. The structural displacement monitoring system is currently being established according to the preferences of researchers or relevant officials. Therefore, a study is required on the optimal displacement monitoring system that can comparatively analyze the displacement measurement data processing methods and that can accurately evaluate the integrity of structures.

This study was conducted to determine the most accurate of the present structure displacement measurement methods that are based on integrated RTK-GPS/accelerometer calculations. The displacement measurement method that is based on integrated RTK-GPS/accelerometer calculation integrates the RTK-GPS and accelerometer displacement measurement methods. Accordingly, the integrated method is influenced by the appropriateness of the two displacement measurement methods and by the method of integrated calculation. RTK-GPS displacement measurements are less affected by their data processing method than are accelerometer displacement measurements. Three methods were used to compare the effects of different acceleration data processing methods: first, the noise within the noise bandwidth is removed by using the same digital filter as that used for the most widely used finite impulse response (FIR) and then double-integration is applied [10]; second, the noise from the acceleration data is removed by using the empirical mode decomposition (EMD) method that was introduced in 1998 [13] and the double-integration is applied; and third, the centered difference method and Tikhonov’s generalization scheme [14] are used without noise filtering for the acceleration data, which were presented by Hong [4]. To compare the effects of the methods of integrating the displacements obtained from the accelerometer and RTK-GPS, two methods were examined: first, the displacements measured from the accelerometer were fitted based on the RTK-GPS displacements, and second, only the dynamic displacements were extracted from the displacements from the accelerometer while the static and quasi-static displacements were extracted from the displacements from RTK-GPS. Cantilever beam specimens were manufactured to ensure rigorous comparison and accuracy evaluation for each displacement measurement method. A linear variable differential transformer (LVDT), accelerometer, and GPS were used to measure the displacements, and multiple displacement types (static, quasi-static, and dynamic) were applied to the specimens. The performance of the displacement measurement methods was evaluated by comparing the displacements from the LVDT with those that were obtained by processing the data from the accelerometer and GPS using each displacement measurement method.

## 2. Methods of Processing Acceleration Data

Two methods of calculating the displacements from the measured acceleration are possible: removing the noise from the acceleration measurement data with a band-pass filter and applying double integration, or using the central differencing scheme and the Tikhonov regularization scheme without additional filtering. In this study, the former method was classified into two methods according to the type of band-pass filter: the case of using the FIR filter (Method A1) and that using the EMD filter (Method A2). We compared these two methods and compared the method of using central differencing and that of Tikhonov regularization (Method A3). The degrees of accuracy of the methods were then analyzed.

### 2.1. FIR Filter and Double Integration: Method A1

The displacements were calculated by removing the noise from the acceleration data using a band-pass filter, and then applying double integration Equation (1), as follows, although the initial value is difficult to determine:

(1) $$s(t)=s_{0}+v_{0}\times t+\int_{0}^{t}\;\left(\int_{0}^{t}\;a(t)\mathrm{dt}\right)\;\mathrm{dt}$$

where *s*(*t*) is the displacement at time *t*, *a*(*t*) is the acceleration at time *t*, *s*_0_ is the initial position, and *v*_0_ is the initial velocity.

**Figure 1.**
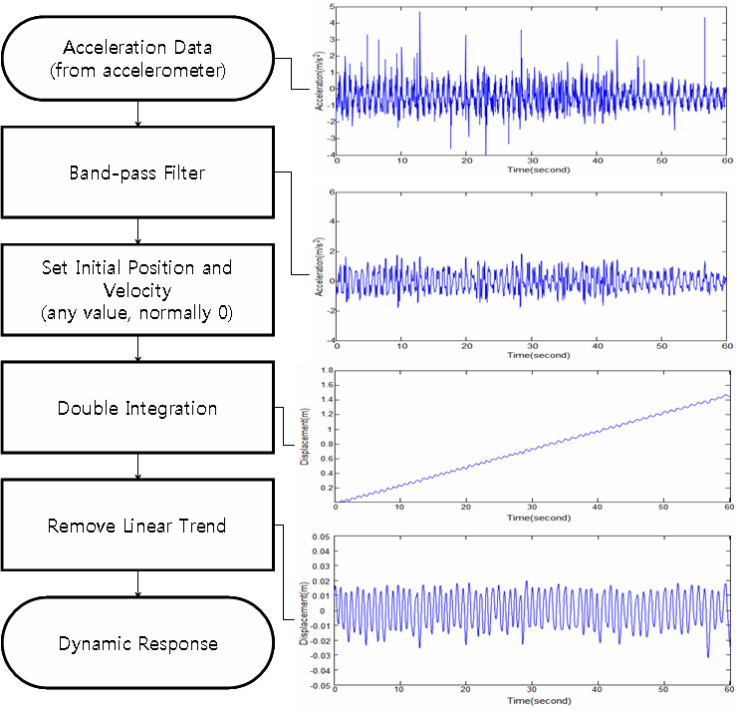
Calculation of the dynamic displacement using the acceleration data.

The initial position (*s*_0_) and the initial velocity (*v*_0_) cannot be measured using the accelerometer, and must be calculated using a separate method. This initial value problem can be solved by applying two conditions. The first condition is that the only displacement that can be measured using the accelerometer is dynamic displacement. According to this condition, static or quasi-static displacement can be measured using other methods, including RTK-GPS. A certain value can be used as the initial position value in processing the accelerometer data. The initial position problem can be addressed by calculating the relative displacement based on a specific value and adjusting it based on the static or quasi-static displacement that is measured using another method. The second condition is that an incorrect initial velocity generates a linear positional error in the calculated displacement. Therefore, the initial velocity value problem can be addressed by calculating the displacement using a specific initial velocity and estimating and removing the linear tendency of the calculated displacement. Figure 1 shows the procedure for calculating the displacement from the acceleration under these conditions.

Digital filters, which are used as band-pass filters for noise filtering, are largely divided into FIR and infinite impulse response (IIR) filters. For choosing the band-pass filter, Method A1 uses the discrete-Fourier-transform (DFT)-based FIR filter, which has a simple structure and ensures stability. The IIR filter is more difficult to design and apply than the FIR filter, but requires a shorter time for calculation. Considering the performance of the recent high-speed processor, however, its advantage cannot justify its replacement of the FIR filter [15].

### 2.2. EMD Filter and Double Integration: Method A2

In order to select the band-pass filter, Method A2 uses an EMD filter. EMD is a new time-frequency analysis method that adaptively and efficiently decomposes the signal, and was introduced by Huang *et al.* [16]. EMD decomposes the signals into sets of functions, which are intrinsic mode functions (IMFs). Each IMF is used as the base function for each frequency. EMD differs from existing signal decomposition methods that use DFT, *etc.*, in that the decomposed functions are not predefined functions, such as the sine function, but are irregular IMFs that have not only frequency characteristics but also the unique characteristics of the original signals. If the original signal is *a*(*t*), the EMD decomposition results can be expressed as in Equation (2). The original signals are divided into N IMFs and residual signals. The lower-order IMFs represent high-frequency signals, and the higher-order IMFs represent low-frequency signals. The residual signals are constants, monotonic slope functions, or functions with only one maximum (or minimum). Equation (2) is as follows:

(2) $$a(t)=\sum_{n=1}^{N}\;C_{n}\;(t)+r_{n}\;(t)$$

where *a*(*t*) is the original signal, *C_n_*(*t*) is the nth IMF, and *r_n_*(*t*) is the residual signal.

The EMD filter is represented by Equation (3) as follows:

(3) $$a_{b}\;(t)=\sum_{n=q}^{r}\;C_{n}\;(t)$$

where 1 < *q* < *r* < *n*.

**Figure 2.**
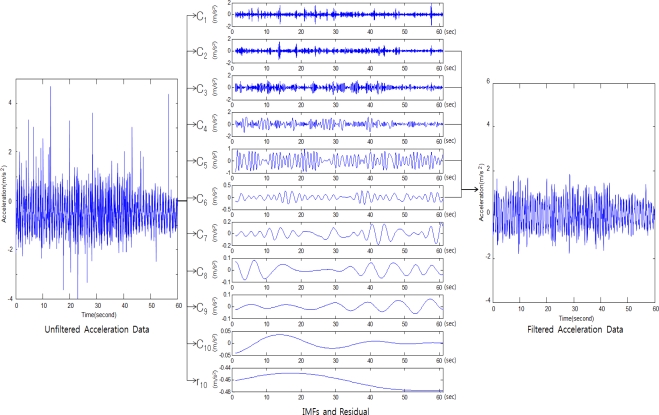
Filtering of the acceleration data using an EMD filter.

In Method A2, the EMD filter was used in the band-pass filtering stage, among the acceleration data processing processes as shown in Figure 1. Figure 2 shows the process of removing the noise by applying the EMD filter to the acceleration data.

### 2.3. Central Differencing and Tikhonov Regularization: Method A3

Hong [4] presented a method that uses central differencing and Tikhonov regularization to calculate the displacements from measurements. By applying central differencing method to the measured acceleration data, the relationship between the acceleration (a), velocity (*v*), and displacement (*s*) can be expressed as Equations (4) and (5):

(4) $$v_{t}=\frac{s_{t+1}-s_{t-1}}{2\Delta t}$$

(5) $$a_{t}=\frac{s_{t+1}-2s_{t}+s_{t-1}}{{\left(\Delta t\right)}^{2}}$$

Equation (5), which shows the relationship between the acceleration and the displacement at a particular point in time, can be extended to the entire time range for n acceleration data to obtain the following matrix equation:

(6) $$\begin{array}{c} \frac{1}{{\left(\Delta t\right)}^{2}}\;\left[\begin{array}{l} 1-2\;\;1\\ \;\;1-2\;\;1\\ \;\;\;\;\;\;\;\;•\\ \;\;\;\;\;\;\;\;\;\;•\\ \;\;\;\;\;\;\;\;\;\;\;\;•\\ \;\;\;\;\;\;\;\;\;\;1-2\;\;1\\ \;\;\;\;\;\;\;\;\;\;\;\;\;\;1-2\;\;1 \end{array}\right]\;\left[\begin{array}{l} s_{0}\\ s_{1}\\ s_{2}\\ •\\ •\\ •\\ s_{n-1}\\ s_{n}\\ s_{n+1} \end{array}\right]=\left[\begin{array}{l} a_{1}\\ a_{2}\\ •\\ •\\ •\\ a_{n-1}\\ a_{n} \end{array}\right]\\ Ls=a \end{array}$$

The matrix Equation (6) provides n equations to find n + 2 unknowns. Due to this rank deficiency, the displacement cannot be calculated using a direct calculation method. Therefore, the Tikhonov regularization scheme must be applied, as follows. Equation (7) can be obtained by applying the regularization scheme. This equation can be used to provide the following equation for calculating the displacement matrix s Equation (8):

(7) $$\mathrm{Min}\;\prod (s)=\frac{1}{2}{\left\|\mathrm{Ls}-\bar{a}\right\|}_{2}^{2}+\frac{\lambda^{2}}{2}{\left\|s\right\|}_{2}^{2}$$

(8) $$\frac{\partial \prod }{\partial s}=0\Rightarrow s={\left(L^{T}\;L+\lambda I\right)}^{-1}\;L^{T}\;a$$

**Figure 3.**
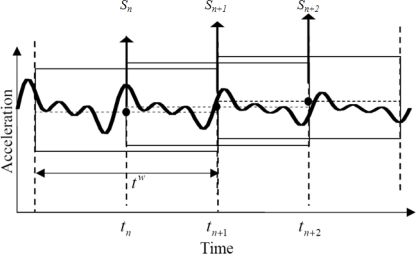
Time windowing technique [4].

λ in Equations (7) and (8) is the generalization coefficient, and is calculated using generalized cross-validation (GCV) method. In this study, the generalization coefficient was determined for harmonic vibration. In Equation (8), a larger number of acceleration measurements leads to a larger matrix and a longer calculation time. Therefore, it is important to choose an n with a proper size to perform repeated sequential calculation. As shown in Figure 3, the time windowing technique can be used, wherein the calculation is repeated for the section with a specific size (*t_w_*), the starting point of the section is increased according to the unit period, and only the medians from the calculation results are used.

## 3. RTK-GPS/Accelerometer Integration

The methods of measuring the overall displacement by integrating the accelerometer displacement measurement (for dynamic structure displacement) and the RTK-GPS displacement measurement (for static and quasi-static displacement) include the RTK-GPS-based simple integration method and the integration that uses frequency-based displacement extraction.

### 3.1. RTK-GPS-Based Simple Integrated Calculation: Method I1

**Figure 4.**
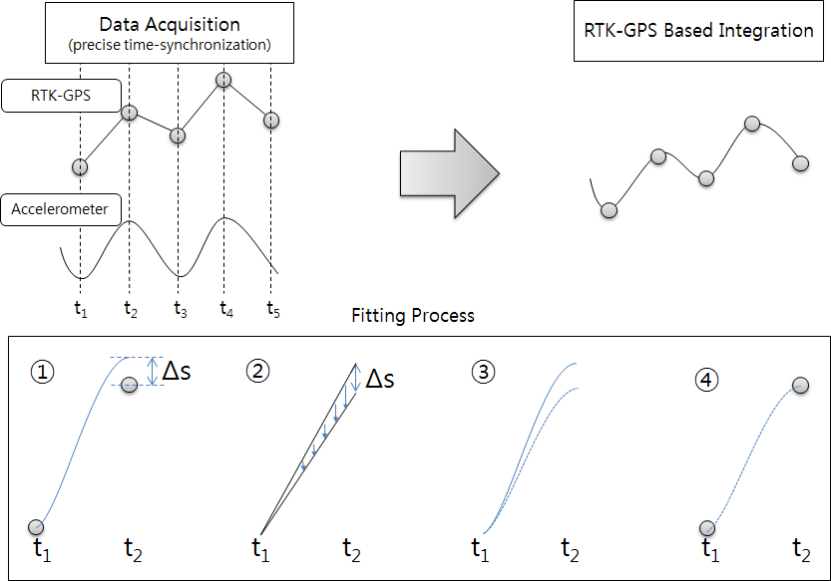
RTK-GPS-based simple integrated calculation process.

The simple integrated calculation visually and precisely synchronizes the measurements from RTK-GPS and the accelerometer, and fits the displacements measured from the accelerometer into the RTK-GPS measurements [17]. Figure 4 shows the concept of the simple integration based on the RTK-GPS measurements.

The integration procedure is as follows. As shown in (1) in Figure 4, the overall displacement between *t*_1_ and *t*_2_ that is measured from the accelerometer is shifted based on the RTK-GPS measurements. At *t*_2_, however, a deviation between the RTK-GPS and accelerometer measurements (Δ*s*) may appear. This deviation can be corrected by calculating the time-based linear deviation tendency (2) and applying the results to the acceleration measurements (3). Thus, the integrated calculation results are obtained (4). The integrated calculation can be performed by applying this integration procedure to all the measurement data. The integrated calculation results include dynamic, static, and quasi-static displacements. Although this integration procedure is simple, it is disadvantageous in that its accuracy depends on the accuracy of the RTK-GPS measurements and it requires precise vision-synchronization processes and devices [18].

### 3.2. Integration by Frequency-Based Displacement Extraction: Method I2

**Figure 5.**
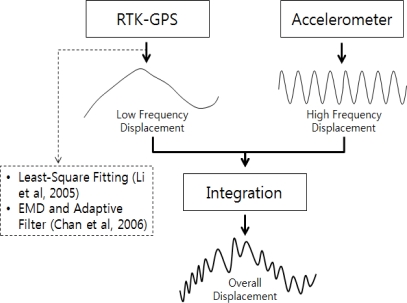
Concept of the integrated calculation according to frequency-based displacement extraction.

The integrated calculation method based on frequency-based displacement extraction was proposed by Li *et al.* [10] and Chan *et al.* [1]. The displacement of a high frequency is extracted from the accelerometer measurements, the displacement of a low frequency is extracted from the RTK-GPS measurements, and the two types of displacement are integrated. Figure 5 shows the concept of the integrated calculation according to frequency-based displacement extraction.

The core part of this integrated calculation is the extraction of the low-frequency bandwidth from the RTK-GPS measurements. Li *et al.* [10] used the least-square fitting method to extract low-frequency displacements. Chan *et al.* [1] used both EMD and an adaptive filter to extract the displacements. In this method, the RTK-GPS measurement displacement is decomposed using EMD to extract IMFs and residual displacements. Then the adaptive filter is applied to the sum of the IMFs, excluding the residual displacements. The displacements that were measured using the acceleration data were taken as the reference values to remove the high-frequency displacements. Finally, the residual displacements were added to the calculated results to include the static, quasi-static, and low-frequency dynamic displacements. Frequency-based displacement extraction can measure the overall structure displacement by adding the high-frequency dynamic displacements that are measured using the acceleration data to the calculated displacements. In this study, the integrated calculation was performed based on frequency-based displacement extraction according to the study results of Chan *et al.* [1], and the results were compared with those of the existing RTK-GPS-based simple integration. The method of Li *et al.* [10] was not used because the test revealed that it cannot accurately extract the low-frequency displacements from the RTK-GPS measurements when quasi-static displacements with relatively abrupt changes appeared.

## 4. Displacement Monitoring Test

**Figure 6.**
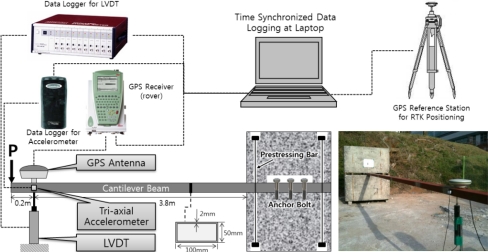
Specimen and devices.

As shown in Figure 6, a cantilever beam bending test was performed to compare the acceleration data processing and displacement measurement methods and to evaluate the effectiveness and accuracy of the RTK-GPS/accelerometer integration methods. This test was selected because it can easily measure the displacements at the free end when an external load is applied to the beam, and can artificially simulate the different displacements that can appear in severe natural environments.

A commercial square-shaped steel pipe was used as the beam in the test. The steel pipe had an ultimate tensile strength of 410 MPa, a yield tensile strength of 240 MPa, and a modulus of elasticity of 200 GPa. A cantilever beam specimen consisting of concrete blocks and a steel pipe beam was manufactured to compare the accuracies of the diverse data processing methods. In addition, LVDT, RTK-GPS, and a low-cost triaxial accelerometer were installed on the beam for the measurement. The test results were stored in a laptop PC after visual time synchronization. Figure 6 shows the specimen, measurement device arrangement, and data collection paths. Also the detailed informations of LVDT and RTK-GPS sensor are summarized in Table 1.

The data for the RTK-GPS measurement were processed on a laptop PC. The time on the laptop PC was constantly updated according to the GPS time to synchronize the time data for the acceleration and LVDT measurements with the GPS time data. The distance between the GPS on the cantilever beam and the base station GPS was maintained at up to 15 m to ensure accurate RTK-GPS measurements. To analyze the accuracy of the three aforementioned acceleration data processing methods and the two integrated RTK-GPS/accelerometer calculation methods, three displacements were given to the cantilever beams, and whether or not the displacements could be accurately calculated was determined using the accelerometer or the integrated RTK-GPS/accelerometer.

**Figure 7.**
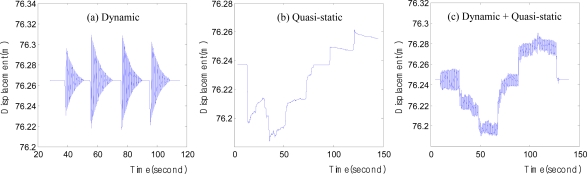
Three types of displacement measurement.

To ensure accurate evaluation, three displacements were generated on the cantilever beam. Among the three displacements shown in Figure 7, the dynamic displacements were generated by the free vibration on the cantilever beam, and the others were artificially generated by the experimenter. This method is highly reliable because LVDT can accurately measure any type of position and displacement, even artificial displacements. Furthermore, the displacement obtained from LVDT can be effectively used as a reference value for evaluating the artificially induced displacement measured using the other measuring devices.

Figure 7(a) includes only the dynamic displacements. This displacement was used for the evaluation and comparative analysis of the degrees of accuracy of the acceleration data processing methods. Figure 7(b) includes only the quasi-static displacements, which were used to analyze the positional measurement of RTK-GPS. Figure 7(c) includes both the dynamic and quasi-static displacements, which were used for the evaluation and comparative analysis of the degrees of accuracy of the integrated RTK-GPS/accelerometer calculation methods. The displacements that were measured using LVDT were used as the reference values for all the accuracy tests. It can be assumed that the evaluation was accurate, as the displacement measurement tolerance of LVDT was less than 0.01 mm.

## 5. Analysis of the Test Results

### 5.1. Accuracy Evaluation via the Acceleration Data Processing Method

Three acceleration data processing methods were used to measure the dynamic displacement of the structure, as described in Section 2. The dynamic displacements in Figure 7(a) were generated at the end of the cantilever beam and based on such displacements the performance levels of the acceleration data processing techniques were evaluated and analyzed.

**Figure 8.**
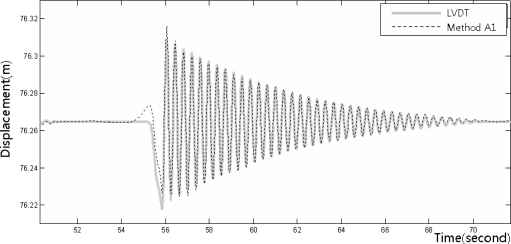
Displacement measurements from the LVDT and the accelerometer (Method A1).

**Figure 9.**
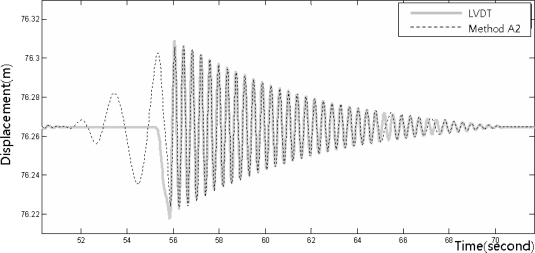
Displacement measurements from the LVDT and the accelerometer (Method A2).

**Figure 10.**
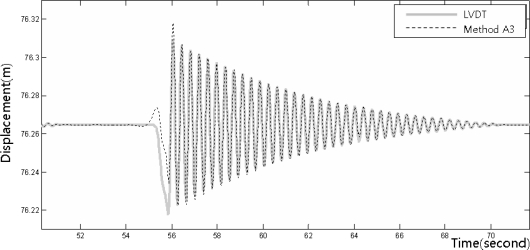
Displacement measurements from the LVDT and the accelerometer (Method A3).

**Figure 11.**
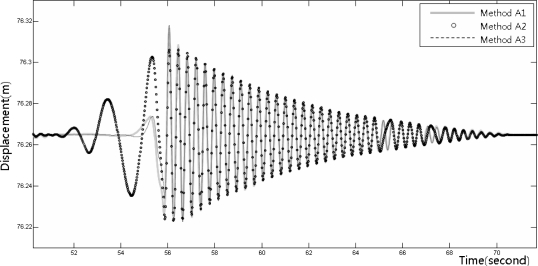
Displacements measured from the accelerometer using the processing method.

Figures 8–10 show the dynamic displacements that were calculated using Methods A1–A3 and the LVDT measurements. The results showed that Method A1 provided the closest calculation results to the actual displacements, and that Method A3 provided the farthest (Figures 8 and 10). In addition, as shown in Figure 9, the results of Method A2 showed excessive or missing displacements in some sections because of insufficient filtering. This may indicate that the filter that was formed in the EMD method, which is suitable for the decomposition of nonlinear signals, was not suitable for removing the noise from the acceleration measurement data. In all the acceleration data processing techniques, a significantly large deviation appeared when the force was applied to generate the displacements; but after the moment, the measurements were very similar to the actual displacements. Figure 11 shows the displacements that were calculated using the acceleration processing methods by superimposing them, except for the LVDT measurements.

Table 2 shows the statistically compared degrees of accuracy of the acceleration data processing methods. Method A1 was the most accurate method. It showed an average deviation of 1 mm. Method A2 showed a deviation of approximately 2 mm, and Method A3 showed a deviation of approximately 4 mm. The maximum errors were 11.9 mm in Method A1, 42.3 mm in Method A2, and 22.6 mm in Method A3. However, these excessive deviations were rare among all the measurements. This was determined from the histogram of the measurement errors according to method.

**Figure 12.**
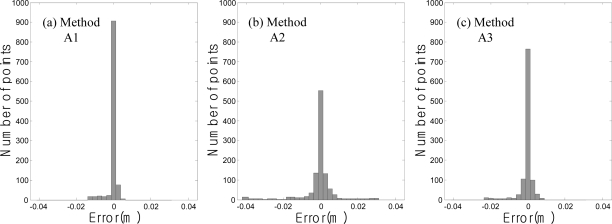
Histogram of the displacement measurement errors using the acceleration data processing method.

Figure 12(a) shows that most of the errors were within ±5 mm and excessive deviations were very rare. As shown in Figure 12(c), this distribution also appeared in Method A3. Relatively significant errors were only predominant in Method A2, as shown in Figure 12(b).

### 5.2. Accuracy Evaluation of the RTK-GPS Displacement Measurement

Prior to the integrated RTK-GPS/accelerometer calculation, the displacement measurement accuracy of RTK-GPS was analyzed. The static and quasi-static displacements of the structure were measured using RTK-GPS, and the accuracy of the RTK-GPS measurement method directly affected the accuracy of the integrated calculation results. The quasi-static displacement that was similar to that in Figure 7(b) was generated at the end of the cantilever beam, and was measured using RTK-GPS. The measurements were compared with the LVDT measurements to evaluate their accuracy. We repeatedly performed the experiment in this way and assessed the accuracy of RTK-GPS.

**Figure 13.**
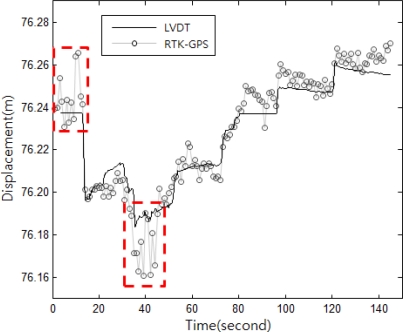
Example of the experiment result for assessing the accuracy of the vertical displacements measured from RTK-GPS, with the distribution of the RTK-GPS measurements with the LVDT values as reference. The dashed rectangles indicate excessive errors.

As shown in Figure 13, the RTK-GPS quasi-static displacement measurements were relatively accurate, but errors of about 20 mm appeared in some sections. Table 3 shows the quantitative analysis results for the RTK-GPS and LVDT measurements. The average deviation was approximately 5 mm, and the maximum error ranged from 20 to 30 mm. This error is similar to the general accuracy of the vertical displacement measurements using RTK-GPS. The vertical coordinate measurement error of the commercial RTK-GPS system is reportedly twice the horizontal coordinate measurement deviation of 10 mm + 1 ppm [19]. The test results showed that RTK-GPS allows displacement measurement within this deviation range.

### 5.3. Accuracy of the Integrated RTK-GPS/Accelerometer Calculation by Method

The two methods that were described in Section 3 were used for the integrated calculation of the RTK-GPS and accelerometer measurements. To comparatively evaluate the degrees of accuracy of these two methods, quasi-static and dynamic displacements were simultaneously generated at the end of the cantilever beam, and their degrees of accuracy were evaluated by comparing the results with the LVDT measurements, as shown in Figure 7(c).

**Figure 14.**
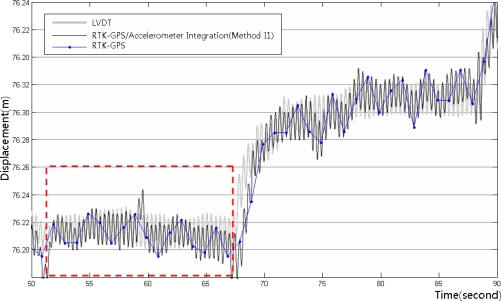
LVDT measurements, RTK-GPS/accelerometer integrated calculation results (Method I1), and long-frequency displacements from the RTK-GPS measurements.

**Figure 15.**
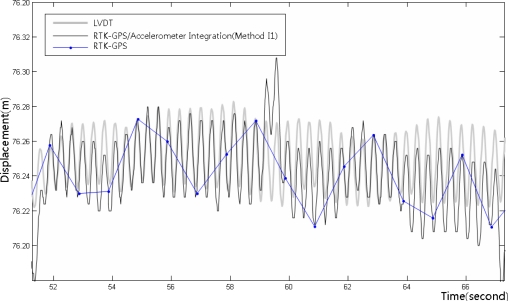
LVDT measurements and integrated RTK-GPS/accelerometer calculation results for the enlarged dashed rectangle area in Figure 14.

Figure 14 shows the integrated calculation results using Method I1, the LVDT measurements, and the RTK-GPS measurements. Figure 15 shows the enlarged dashed rectangle area in Figure 14. These figures indicate that the RTK-GPS measurements are relatively accurate with respect to the quasi-static displacements that were generated in the test, but not with respect to the dynamic displacements that were generated at a 3 Hz frequency due to the limitation in the measurement frequency. In the integrated calculation using Method I1, the size and frequency of the calculated dynamic displacements were very similar to those of the LVDT measurements, but they generally showed 1 to 2 cm deviations. In addition, on the right side of Figure 15, the LVDT measurements show relatively gentle quasi-static displacements, but the integrated calculation results fluctuate somewhat irregularly. This occurred due to the limitation in the RTK-GPS measurement accuracy and the characteristic dependency of the integrated calculation results on the RTK-GPS measurement in Method I1. Thus, the results of the RTK-GPS/accelerometer integration using Method I1 were relatively accurate with respect to the dynamic and quasi-static displacements, but they involved 1 to 2 cm deviations and were dependent on the RTK-GPS measurements.

**Figure 16.**
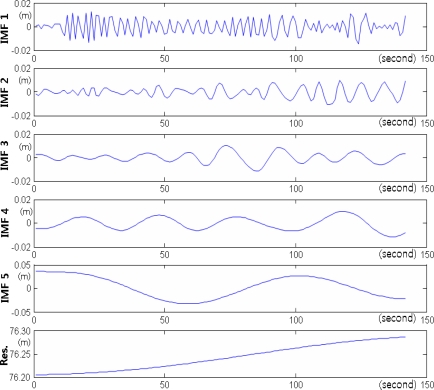
RTK-GPS measurements decomposed by frequency using the EMD method. The intrinsic mode functions (IMFs) 1–5 and one residual were generated.

In the integrated calculation using Method I2, the low-frequency displacements were extracted from the RTK-GPS measurements, and were then integrated with the high-frequency displacements that were measured using an accelerometer. First, the RTK-GPS measurements were decomposed by frequency using the EMD method, as shown in Figure 16, and the residual displacements were removed to extract the low-frequency displacements from the RTK-GPS measurements. An adaptive filter was then used, with the accelerometer displacements as the reference values, to extract the low-frequency displacements. Finally, the extracted low-frequency displacements were integrated with the residual displacements to calculate the final displacements.

**Figure 17.**
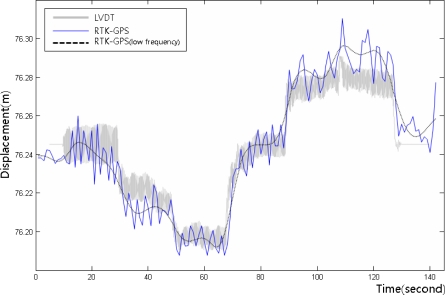
LVDT and RTK-GPS measurements and low-frequency displacements extracted from the RTK-GPS measurements.

Figure 17 shows the LVDT and RTK-GPS measurements and the final low-frequency RTK-GPS measurements. The low-frequency RTK-GPS measurements should ideally be similar to the center line of the LVDT measurements, but 15 mm deviations appeared in some sections due to the limitation in the vertical displacement measurement accuracy of RTK-GPS.

EMD was the only method used to extract the high-frequency displacements from the acceleration data. After the acceleration data were decomposed into IMF functions and residuals in the EMD method, the IMF functions, except for one low-frequency IMF and residual, were summarized to extract the high-frequency displacements.

**Figure 18.**
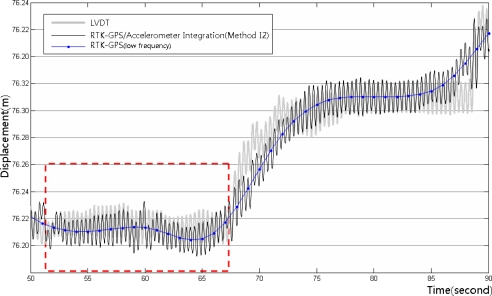
LVDT measurements, RTK-GPS/accelerometer integrated calculation results (Method I2), and low-frequency displacements from the RTK-GPS measurements.

**Figure 19.**
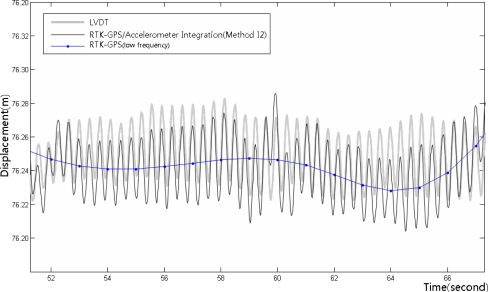
LVDT measurements and integrated RTK-GPS/accelerometer calculation results for the enlarged dashed rectangle area in Figure 18.

Figure 18 shows the integrated RTK-GPS/accelerometer displacements (Method I2), LVDT measurements, and low-frequency RTK-GPS measurements. Figure 19 shows the enlarged dashed rectangle area in Figure 18.

Figures 18 and 19 show that both the dynamic and the quasi-static displacements are accurate. The measurements were accurate in the sections wherein the slope of the quasi-static displacements was gentle, contrary to the simple integrated calculation. The results were more accurate than those of the simple integrated calculation, but the measurement was somewhat inaccurate when the quasi-static displacements abruptly appeared.

**Figure 20.**
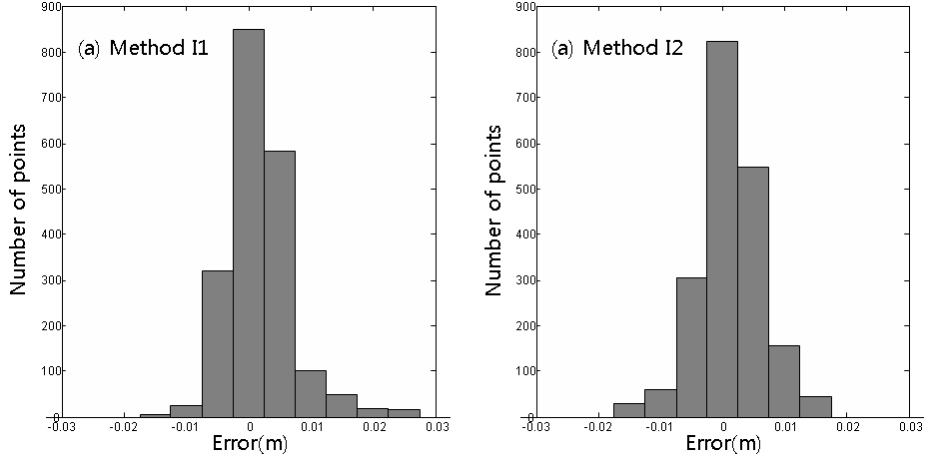
Histogram of the integrated RTK-GPS/accelerometer calculation error by method.

Table 4 shows the statistical analysis of the accuracy of the calculation results from Methods I1 and I2. The average deviations and standard deviations were similar at less than 5 mm in both methods, but the frequency-based displacement extraction was more accurate for the maximum deviation and the less-than-5 mm deviations. As shown in the distribution of the measurement deviations in the integrated calculation method in Figure 20, both methods mostly showed low deviations. The deviation distribution of the measurement results using Method I2 (frequency-based displacement extraction) was superior to that with Method I2.

## 6. Conclusions

In this study, diverse acceleration data processing methods and integrated calculation methods were evaluated to determine an optimal integrated RTK-GPS/accelerometer calculation method for structure displacement measurement. A cantilever beam bending test was conducted to evaluate the accuracy and usefulness of acceleration data processing methods and integrated calculation methods. For the evaluation, the LVDT measurements were directly compared with the results of the displacement estimation methods.

1. The accuracies of the three displacement calculation methods that used acceleration data processing were comparatively analyzed. The accuracy was highest when an FIR band-pass filter and double integration were used for the calculation, and it was also high when central differencing and Tikhonov regularization were used. This indicates that the displacement calculation methods can be used to measure the dynamic displacement with an accelerometer. The method with the EMD filter and double integration can also be used, but its accuracy was inferior to that of the aforementioned two methods.
2. The quasi-static displacement in the structure was measured using the RTK-GPS method. The displacements were measured with 5 mm deviations on average at a maximum error of 30 mm.
3. Among the integrated RTK-GPS/accelerometer calculation methods, the RTK-GPS-based simple integrated calculation and the frequency-based displacement extraction measured both the dynamic and quasi-static displacements. The test results showed that the latter was more accurate, with 4 mm deviations on average at a maximum error of 20 mm.
4. Referencing the results of the test conducted in this study, it was found that precise and overall measurement of the multiple structural displacements was possible using the optimal RTK-GPS/accelerometer integration method, which was used in this study.
5. For further study, an experiment involving the application of the optimal method to a real structure will be conducted to verify the benefits of the method.

The accuracy-related values in the conclusion of this study are experimental values, and cannot be considered absolutely accurate due to the characteristics of the RTK-GPS method that are affected by the GPS satellite signal reception environment. Further studies and tests are required to present more general and stricter standards.

## Figures and Tables

**Table 1.** Key informations of LVDT transducer and RTK-GPS surveying.

**LVDT**		**RTK-GPS**	
Items	Specification	Items	Specification
Type	CDP-100 (tokyo sokki kenkujo)	Used software	RTKNavTM (Novatel Inc.)
Capacity	100 mm	GPS receiver/antenna	GX1200 with PPS/Event option (Leica Geosystems Inc.)/LEIAX1202GG (Leica Geosystems Inc.)
Rated output	5 mV/V ± 0.1% (10,000 × 10^−6^ strain ± 0.1%)	GPS antenna calibration	Antenna calibration information from national geodetic survey of USA was used
Sensitivity	100 × 10^−6^ strain/mm	GPS base station position	Previously determined by static GPS surveying method referred to national GPS station
Nonlinearity	0.1% RO	Reference frame (ellipsoid)/Positioning method	ITRF 2000 (GRS 1980)/Real time kinematic positioning (using RTCM 2.3 correction message)
Temperature range	−10∼+60 °C	Ephemeris/Positioning frequency	Broadcasting ephemeris/1 Hz

**Table 2.** Accuracy using the acceleration data processing method.

**Statistics index**	**Method A1**	**Method A2**	**Method A3**
Maximum deviation (m)	0.0119	0.0423	0.0226
Mean deviation (m)	0.0011	0.0041	0.0018
Standard deviation (m)	0.0023	0.0085	0.0037
Ratio of less than 5 mm deviation (%)	94.4	81.9	93.4

**Table 3.** Accuracy of the vertical displacements measured with RTK-GPS.

**Statistics Index**	**Experiment Results**
Maximum deviation (m)	0.0253
Mean deviation (m)	0.0057
Standard deviation (m)	0.0076
Ratio of less than 20 mm deviation (%)	96.5

**Table 4.** Accuracy of the integrated RTK-GPS/accelerometer calculation according to method.

**Statistics Index**	**Method I1**	**Method I2**
Maximum deviation (m)	0.0273	0.0153
Mean deviation (m)	0.0037	0.0037
Standard deviation (m)	0.0053	0.0050
Ratio of less than 5 mm deviation (%)	89.2	91.2
